# Polygenic Analysis of Late-Onset Alzheimer’s Disease from Mainland China

**DOI:** 10.1371/journal.pone.0144898

**Published:** 2015-12-17

**Authors:** Bin Jiao, Xiaoyan Liu, Lin Zhou, Maggie Haitian Wang, Yafang Zhou, Tingting Xiao, Weiwei Zhang, Rui Sun, Mary Miu Yee Waye, Beisha Tang, Lu Shen

**Affiliations:** 1 Department of Neurology, Xiangya Hospital, Central South University, Changsha, China; 2 State Key Laboratory of Medical Genetics, Changsha, China; 3 Key Laboratory of Hunan Province in Neurodegenerative Disorders, Central South University, Changsha, China; 4 Division of Biostatistics, School of Public Health and Primary Care, the Chinese University of Hong Kong, Shatin, Hong Kong Special Administrative Region; 5 School of Biomedical Sciences, the Chinese University of Hong Kong, Shatin, Hong Kong Special Administrative Region; Cleveland Clinic Foundation, UNITED STATES

## Abstract

Recently, a number of single nucleotide polymorphisms (SNPs) were identified to be associated with late-onset Alzheimer disease (LOAD) through genome-wide association study data. Identification of SNP-SNP interaction played an important role in better understanding genetic basis of LOAD. In this study, fifty-eight SNPs were screened in a cohort of 229 LOAD cases and 318 controls from mainland China, and their interaction was evaluated by a series of analysis methods. Seven risk SNPs and six protective SNPs were identified to be associated with LOAD. Risk SNPs included rs9331888 (*CLU)*, rs6691117 (*CR1)*, rs4938933 (*MS4A)*, rs9349407 (*CD2AP)*, rs1160985 (*TOMM40)*, rs4945261 (*GAB2)* and rs5984894 (*PCDH11X)*; Protective SNPs consisted of rs744373 (*BIN1)*, rs1562990 (*MS4A)*, rs597668 (*EXOC3L2)*, rs9271192 (*HLA-DRB5/DRB1)*, rs157581 and rs11556505 (*TOMM40)*. Among positive SNPs presented above, we found the interaction between rs4938933 (risk) and rs1562990 (protective) in *MS4A* weakened their each effect for LOAD; for three significant SNPs in *TOMM40*, their cumulative interaction induced the two protective SNPs effects lost and made the risk SNP effect aggravate for LOAD. Finally, we found rs6656401-rs3865444 (*CR1-CD33*) pairs were significantly associated with decreasing LOAD risk, while rs28834970-rs6656401 (*PTK2B-CR1*), and rs28834970-rs6656401 (*PTK2B-CD33*) were associated with increasing LOAD risk. In a word, our study indicates that SNP-SNP interaction existed in the same gene or cross different genes, which could weaken or aggravate their initial single effects for LOAD.

## Introduction

Alzheimer’s disease (AD) is a clinically complex neurodegenerative disorder, affecting up to 81.1 million people worldwide [1]. It is characterized by memory and other cognitive decline, a variety of neuropsychiatric symptoms and restriction in the activities of daily living [2]. According to the age of onset, AD was classified as early-onset AD (EOAD, equal or less than 65 years) and late-onset AD (LOAD, more than 65 years), while the latter is the most common type of AD. The pathogenesis of AD is complicated, mainly caused by genetics, environment and normal aging [3]. To date, there are still only three causative genes reported in familial EOAD patients, including presenilin 1 (*PSEN1*), presenilin 2 (*PSEN2*) and amyloid precursor protein (*APP*) genes [4]. As the majority of patients were LOAD, many genetic association studies have been conducted in recent years to uncover the genetic contributions to LOAD, but the only gene variant considered to be an established LOAD risk factor was the *APOEε4* allele [5].

However, *APOE* was limited to account for approximately 50% of individuals with LOAD [6], indicating other genes could contribute to LOAD risk. Until now, at least two methods are suggested to identify the risk gene variants for LOAD, including Whole Exome Sequence (WES) and genome-wide association study (GWAS) [7,8]. The former is mainly to identify rare coding variants, such as rs75932628 in *TREM2*, rs145999145 in *PLD3* and rs137875858 in *UNC5C*, which were recently recognized as risk variants for LOAD [7,9–11]. With regard to common variants for AD, since 2009, five large GWAS and one Meta-analysis have identified more than 20 loci significantly associated with LOAD. According to the possible role in the process of AD, these genes were classified as several groups: (1) Lipid metabolism: *APOE CLU*, *ABCA7*, SORL1; (2) Immune response: *CR1*, *CD33*, *MS4A*, *EPHA1*, *CLU*, *ABCA7*, HLA-DRB5/DRB1 and INPP5D; (3) Endocytosis: *BIN1*, *PICALM*, *CD2AP*, *EPHA1*, *RIN3*, *SORL1*, *MEF2C* and *MADD* [8,12–16].

However, in most cases, the identified single nucleotide polymorphisms (SNPs) have small or moderate effect sizes, and the proportion of heritability explained is quite modest. Like other complicated diseases, a polygenic analysis has been suggested to explain genetic contribution to the pathogenesis of the majority of LOAD cases. We hypothesized that SNPs might interact in subtle ways that led to substantially greater effects than the effect of any single SNP. Therefore, in this study, we adopted a series of statistical analysis methods to evaluate SNP-SNP interactions in a Chinese cohort consisting of 547 individuals. It was found that SNP-SNP interaction could weak or aggravate their single effect for LOAD. In addition, even if some variants had no effect on LOAD, their interactions could become significant effects associated with LOAD.

## Materials and Methods

### Sample subjects

A total of 547 subjects were recruited in this study, including 229 patients with LOAD (male 43.1%; age at onset: 75.2±5.0 years) and 318 controls (male 47.8%; age:71.6±2.5 years). All patients diagnosed as probable or definite AD met with the NINCDS-ADRDA criteria, and they were collected from the department of neurology, Xiangya Hospital. Analyses also included all unaffected individuals of matched geographical ancestry as healthy controls. The study was approved by the Ethics Committee of Xiangya Hospital, Central South University in China (equivalent to an Institutional Review Board). Written informed consent was obtained from all subjects (if the patient was no capacity to understand this study due to cognitive impairment, written informed consent was obtained from their legal guardians).

### Genotyping methods

Genomic DNA was extracted from peripheral blood leukocytes of all subjects. We used a fluorometer to evaluate the quality and quantity of DNA, which were normalized to 50 ng/μl for sequencing. APOE genotype was identified through polymerase chain reaction (PCR), and the primer information was listed in Table A in S1 File. We sequenced all PCR products with BigDye terminator v3.1 sequencing chemistry and performed on an ABI 3730xl DNA analyzer (Applied Biosystems). Finally, the DNA sequences were read by Sequencher software.

We then selected a list of AD candidate genes based on published GWAS paper and Alzgene.org (http://www.alzgene.org/) [8,12–16]. Totally, 56 SNPs in 28 candidate genes were selected in this study, and the genotypes of 547 subjects were screened using MALDI-TOF mass array method. To identify the accuracy of this method, *APOE* genotypes (rs7412 and rs429358) were also screened, and the result of this method was consistent with that of Sanger sequencing, The 56 SNPs involved *BIN1* (rs744373, rs7561528, rs6733839), *CLU* (rs2279590, rs11136000, rs1532278, rs9331888, rs9331896), *ABCA7* (rs3764650, rs4147929), *CR1* (rs6656401, rs3818361, rs6701713, rs6691117), *PICALM* (rs3851179, rs592297, rs541458, rs561655, rs10792832), *MS4A* (rs4938933, rs670139, rs1562990, rs983392), *CD33* (rs3865444, rs3826656, rs12459419), *CD2AP* (rs9349407, rs10948363), *EPHA1* (rs11767557, rs11771145), *TOMM40* (rs2075650, rs157581, rs157580, rs8106922, rs11556505, rs1160985), *LRAT* (rs727153), *TNK1* (rs1554948), *ARID5B* (rs2588969), *GAB2* (rs10793294, rs4945261), *PCDH11X* (rs5984894) *SORL1*(rs11218343, rs668387) *PTK2B* (rs28834970), *ATP7B* (rs1801243), *EXOC3L2* (rs597668), *SLC24A4-RIN3* (rs10498633), *ZCWPW1* (rs1476679), *CELF1* (rs10838725), *MEF2C* (rs190982), *NME8* (rs2718058), *HLA-DRB5/DRB1* (rs9271192), *FERMT2* (rs17125944), *ECE1* (rs213045) and *CASS4* (rs7274581) (Table A in S2 File).

### Statistical methods

Hardy-Weinberg equilibrium was analyzed using SHEsis software [17]. Logistic regression analysis was used to test for associations between each SNP allele and LOAD risk after adjusting age and gender. We then put all positive SNPs into multivariable logistic regression model to evaluate the association between each SNP and LOAD susceptibility. All above statistics were analyzed using SPSS 17.0 version. The p value<0.05 was defined as statistical significance.

Finally, Lasso-multiple regression (LMR) method was used to identify SNP-SNP pairs’ interaction. The calculation involved two steps: In step one, the Lasso was used to select candidate SNPs with all pair-wise interaction terms in the regression model, which was conducted under the R package glnetmi. Ten-fold cross validation was performed in this step. Interaction terms were ranked by the absolute value of the coefficients from high to low. Thus, it could achieve the aim of feature selection. In the second step, multiple linear regression was performed on the candidate SNPs selected in the first step to evaluate the pure interaction effect. The variable significance was measured by the p-value of cross-product term. Stepwise AIC selection was applied on the multiple linear regression to obtain an optimal regression model [18–20].

## Results

The frequencies of *APOE* alleles and genotypes of all subjects were listed in Table 1. The distribution of ε4 allele in LOAD cases was significantly higher than that of controls (p = 0.002), and lower than that of controls for ε2 allele (p = 0.009). With regard to genotype, there was a significant difference in ε3/4 and ε4/4 genotypes between them (p = 0.004, p = 0.047) (Table 1).

**Table 1:** The distribution of APOE genotypes and alleles in cases and controls.

APOE	Case (n = 229, %)	Control (n = 318, %)	P
ε2	22 (4.8)	57 (9.0)	0.009
ε3	355 (77.5)	509 (80.0)	0.313
ε4	81 (17.7)	70 (11.0)	0.002
ε2/2	0	5 (1.6)	0.078
ε2/3	18 (7.9)	35 (11.0)	0.220
ε2/4	4 (1.7)	12 (3.8)	0.204
ε3/3	139 (60.7)	212 (66.7)	0.151
ε3/4	59 (25.8)	50 (15.7)	0.004
ε4/4	9 (4.0)	4 (1.3)	0.047

All candidate SNPs were in Hardy-Weinberg equilibrium except rs2075650 and rs7274581, therefore, we deleted both of them in the further analysis. Totally, 13 significantly different allelic frequencies were identified between patients and controls after adjusting age and gender, including seven risk SNPs and six protective SNPs for LOAD. The former consisted of rs9331888-C in *CLU*, rs6691117-A in *CR1*, rs4938933-C in *MS4A*, rs9349407-C in *CD2AP*, rs1160985-C in *TOMM40*, rs4945261-A in *GAB2*, rs5984894-A in *PCDH11X*, and the protective SNPs involved rs744373-T in *BIN1*, rs1562990-C in *MS4A*, rs597668-T in *EXOC3L2*, rs9271192-C in *HLA-DRB5/DRB1*, rs157581-G and rs11556505-T in *TOMM40* (Table 2). To explore whether the identified seven risk SNPs or six protective SNPs had an interaction among them, we further analyzed them using multivariable logistic regression model. Finally, we found eleven SNPs had an independent effect for LOAD, except rs1160985 and rs157581 after adjusting age, gender and *APOE* ε4. (Tables A and B in S3 File).

**Table 2:** Independent association of the 54 SNPs with LOAD.

SNP	Gene	Allele	OR (95%CI)	P
rs744373	BIN1	C/T	0.678 (0.526–0.874)	0.003
rs7561528	BIN1	A/G	0.897 (0.630–1.277)	0.546
rs6733839	BIN1	C/T	1.136 (0.890–1.451)	0.306
rs2279590	CLU	A/G	0.844 (0.618–1.152)	0.284
rs11136000	CLU	C/T	1.112 (0.826–1.511)	0.473
rs1532278	CLU	C/T	1.117 (0.828–1.507)	0.468
rs9331888	CLU	C/G	1.328 (1.044–1.690)	0.021
rs9331896	CLU	T/C	1.112 (0.823–1.502)	0.490
rs3764650	ABCA7	G/T	1.044 (0.809–1.349)	0.740
rs4147929	ABCA7	A/G	1.095 (0.853–1.407)	0.476
rs6656401	CR1	A/G	0.900 (0.443–1.829)	0.770
rs3818361	CR1	C/T	0.908 (0.708–1.163)	0.444
rs6701713	CR1	A/G	1.140 (0.889–1.462)	0.302
rs6691117	CR1	A/G	1.481 (1.138–1.926)	0.003
rs3851179	PICALM	A/G	0.902 (0.704–1.155)	0.413
rs592297	PICALM	C/T	1.272 (0.993–1.631)	0.057
rs541458	PICALM	C/T	1.086 (0.854–1.381)	0.502
rs561655	PICALM	A/G	0.983 (0.772–1.250)	0.886
rs10792832	PICALM	A/G	0.894 (0.700–1.144)	0.373
rs4938933	MS4A	C/T	1.350 (1.041–1.751)	0.023
rs670139	MS4A	A/C	0.849 (0.665–1.084)	0.189
rs1562990	MS4A	A/C	0.658 (0.514–0.842)	0.001
rs983392	MS4A	A/G	1.185 (0.750–1.873)	0.467
rs3865444	CD33	G/T	1.110 (0.834–1.478)	0.475
rs3826656	CD33	A/G	0.839 (0.648–1.086)	0.182
rs12459419	CD33	C/T	1.081 (0.811–1.441)	0.593
rs9349407	CD2AP	C/G	1.368 (1.002–1.867)	0.048
rs10948363	CD2AP	A/G	1.138 (0.844–1.535)	0.395
rs11767557	EPHA1	C/T	1.088 (0.783–1.511)	0.614
rs11771145	EPHA1	A/G	0.947 (0.744–1.204)	0.655
rs157581	TOMM40	A/G	0.682 (0.520–0.894)	0.005
rs157580	TOMM40	A/G	1.098 (0.863–1.397)	0.449
rs8106922	TOMM40	A/G	1.179 (0.865–1.607)	0.297
rs11556505	TOMM40	C/T	0.482 (0.340–0.685)	<0.001
rs1160985	TOMM40	C/T	1.319 (1.007–1.728)	0.044
rs727153	LRAT	A/G	0.996 (0.753–1.317)	0.978
rs1554948	TNK1	A/T	0.922 (0.701–1.213)	0.563
rs2588969	ARID5B	A/C	0.861 (0.676–1.096)	0.224
rs10793294	GAB2	A/C	0.775 (0.577–1.040)	0.089
rs4945261	GAB2	A/G	1.308 (1.026–1.666)	0.030
rs5984894	PCDH11X	A/G	1.934 (1.380–2.712)	<0.001
rs11218343	SORL1	C/T	0.939 (0.707–1.247)	0.664
rs668387	SORL1	A/G	0.819 (0.641–1.047)	0.111
rs28834970	PTK2B	C/T	0.827 (0.634–1.079)	0.162
rs1801243	ATP7B	G/T	0.986 (0.776–1.254)	0.909
rs597668	EXOC3L2	C/T	0.774 (0.604–0.991)	0.042
rs9271192	HLA-DRB5/DRB1	A/C	0.703 (0.521–0.949)	0.021
rs10498633	SLC24A4-RIN3	G/T	1.164 (0.782–1.732)	0.455
rs1476679	ZCWPW1	C/T	1.168 (0.898–1.519)	0.246
rs10838725	CELF1	C/T	0.842 (0.650–1.090)	0.192
rs2718058	NME8	A/G	1.245 (0.920–1.685)	0.155
rs17125944	FERMT2	C/T	0.967 (0.728–1.285)	0.818
rs190982	MEF2C	A/G	1.047 (0.747–1.461)	0.798
rs213045	ECE-1	G/T	1.206 (0.947–1.535)	0.130

It was noted that both risk and protective SNPs were identified in *MS4A* (rs4938933-risk and rs1562990-protective) and *TOMM40* (rs1160985-risk, rs157581-protective, and rs11556505-protective). With regard to two SNPs in *MS4A*, a total of four subhaplotypes were grouped, while no significant difference was identified between cases and controls (Table 3). In addition, eight subhaplotypes were combined within three SNPs in *TOMM40*, and the only subhaplotype G-T-C (rs157581-rs11556505-rs1160985) was significantly susceptible to LOAD risk. (Table 4)

**Table 3:** Frequencies of subhaplotypes (rs4938933-rs1562990*) in MS4A.

Subhaplotypes	Case (%)	Control (%)	P	OR (95%CI)
C-A	5.8	6.8	0.519	0.849 (0.516–1.397)
C-C	26.9	21.8	0.051	1.320 (0.998–1.746)
T-A	52.5	57.7	0.088	0.810 (0.636–1.032)
T-C	14.8	13.7	0.618	1.091 (0.774–1.537)

* rs493893 was risk SNP for LOAD; rs1562990 was protective SNP for LOAD.

**Table 4:** Frequencies of subhaplotypes in TOMM40*.

Subhaplotypes**	Case (%)	Control (%)	P	OR (95%CI)
A-C-C	51.0	55.0	0.219	0.849 (0.675–1.094)
A-C-T	19.7	20.3	0.834	1.320 (0.717–1.308)
G-C-C	4.9	4.7	0.881	0.810 (0.596–1.829)
G-C-T	7.4	9.0	0.370	0.817 (0.524–1.272)
G-T-C	16.5	11.0	0.007	1.611 (1.135–2.286)

* rs157581 (protective SNP)-rs11556505 (protective SNP)-rs1160985 (risk SNP);

** We deleted the combination of G-T-T, A-T-C and A-T-T, due to the low frequency in each group (<0.03).

We further used LMR method to analyze SNP-SNP pairs’ interaction: in the first step, 20 SNPs were selected as candidates from 56 SNPs (Tables A and B in S4 File); in the second step, three SNP-SNP pairs were found to be statistically significant after adjusting multiple testing by Bonferroni correction (Table 5). Three SNP pairs were identified to be significantly associated with LOAD (adjusted p-value to be 0.017, 0.018 and 0.032), including rs6656401-rs3865444 (*CR1*-*CD33*), rs28834970-rs6656401 (*PTK2B*-*CR1*), rs28834970- rs6656401 (*PTK2B-CD33*). The pair (rs6656401-rs3865444) with combination (AG, GT) reduced the LOAD risk by 0.10 compared to (GG, GG), while the other two SNP pairs had an increase risk for LOAD. The pair (rs28834970-rs6656401) with genotype (CT, AG) increased the LOAD risk by 10.90 compared to (TT, GG) and genotype (CT, GT) on (rs28834970-rs3865444) had an increased risk by 1.54 compared to (TT, GG).

**Table 5:** The significant pairs were detected using LMR method.

No.	SNP1	SNP2	p-value*	Adjusted p-value	OR(95%CI)
1	6656401	3865444	0.87 X10^-4^	0.017	0.10 (0.01–0.89)
2	28834970	6656401	0.93 X10^-4^	0.018	10.90 (2.31–51.46)
3	28834970	3865444	1.68 X10^-4^	0.032	1.54 (1.00–2,37)

*: p-values before bonferroni correction

## Discussion

LOAD was one of common diseases with strong genetic component [3]. Although *APOE* ε4 explained a portion of LOAD, other loci, especially identified through GWAS of LOAD, probably also participated in the process of LOAD. [21,22]. Researching these variants will lead to better understanding its biological function that can help in LOAD risk assessment, diagnosis and development of new therapies for LOAD. In this study, we conducted a comprehensive analysis of 56 SNPs using univariate analysis, multiple logistic regression and LMR methods. To our knowledge, it was the most comprehensive genetic analysis for LOAD patients from mainland China.

In current study, we found seven risk SNPs in *CLU*, *CR1*, *MS4A*, *CD2AP*, *TOMM40*, *GAB2* and *PCDH11X* genes conferred to LOAD risk. *CLU*, known as apolipoprotein J probably increased AD risk through interacting with *APOE* [23]. A Meta-analysis showed rs9331888 was significantly associated with AD risk in Caucasian population [24], and we successfully replicated this risk loci. The role of *CR1* in AD development has been highlighted due to involving in erythrocyte amyloid β42 sequestration and clearance of Aβ from whole blood [25], and the missense variant rs6691117 (Ile→Val) may change the folding of CR1 protein and affect structural stability through affecting Hsp70 binding, then cause functional changes [26]. The third identified risk gene was *MS4A*, which may be associated with the control of intracellular free Ca2+ concentration, resulting in neuronal death and decline in cognition [27]. The SNP rs4938933-C in *MS4A* was previously reported to be associated with decreasing LOAD risk in white population [15], while our result displayed rs4938933-C was a susceptible loci for LOAD risk. *CD2AP* was probably linked to modulating amyloid β clearance and tau neurotoxicity [28]. In this study, we first reported the SNP rs9349407 in *CD2AP* was significantly associated with LOAD in Chinese Han population, which was first identified to be significantly associated with AD in European ancestry, while the recent studies failed to replicate this result in Japanese, African-American and Canadian [29]. The *TOMM40* gene is located adjacent to *APOE*. These two proteins may interact with each other to affect mitochondrial dynamics, although the precise mechanism underlying this is unclear. An interesting study showed the SNP rs1160985 was found to be associated with serum triglyceride concentration [30]. *GAB2* is well characterized as a risk gene for the development of AD, which probably interacts with *APOE* ε4 to further modify risk [31]. With regard to *PCDH11X*, the detail of its biological mechanism was unclear, which needs us do more research to know about their relation with LOAD.

Until now, only two variants were identified to protect individuals away from AD: rs63750847-A in *APP* and *APOE*ε2 genotype [32]. However, these two genes were causative or risk for AD. Therefore, we hypothesized some risk genes might also carry some protective variants. In current study, six SNPs were found to be associated with decreasing LOAD risk in *BIN1*, *TOMM40*, *MS4A*, *EXOC3L2* and *HLA-DRB5/DRB1*. However, among them, five SNPs were previously identified risk loci except rs1562990-C in MS4A. Our result indicated that the impact of susceptible genes varied in different ethnicities, which could help us better understand the contributions of genetics to LOAD from Chinese Han population. In addition, more samples should be confirmed these protective variants.

No matter risk SNPs or protective SNPs, further analysis using multivariable logistic regression indicated most of significant loci might have an independent effect on LOAD, which confirmed AD was one of multifactorial diseases with great complex genetic background. Meanwhile, due to our positive result including different genes, it is worth to be focused on exploring their relation between amyloid-β pathology or tauopathy and these genes functions (lipid metabolism, immune response and endocytosis) in the future.

Another goal for current study was to identify whether SNP-SNP interactions existed in the pathogenesis of LOAD. We first analyzed the interactions between positive SNPs in the same gene. For rs4938933 and rs1562990 in *MS4A*, although they had independent reverse effect on LOAD, both of their respective effects were loss when being considered together. With regard to rs157581, rs11556505 and rs1160985 in *TOMM40*, although they were involved in decline or increasing risk for LOAD, the only significant subhaplotype was G-T-C that could increase LOAD risk, which indicates the SNPs effect on LOAD could be influenced by other SNPs in the same gene.

In addition, we further used LMR analysis method to identify three SNP pairs significantly associated with LOAD risk. However, the significant effect was not to be found in any of them during the previous univariate analysis. Among them, one pair rs28834970-rs6656401 from *PTK2B*-*CR1* gene should be concerned due to the high Odds Ratio score (10.90) for LOAD risk, suggesting these two genes may have an strong interaction effect in the pathogenesis pathway. Previous study has identified two significant SNP-pairs for LOAD risk: rs386544-rs670139 from *CLU-MS4A4E* and rs11136000-rs670139 from *CD33-MS4A4E*. Taken these results together presented above, we offered evidence that SNP-SNP interaction played a pivotal role in LOAD susceptibility. As we know, AD is a complicated disease with gene-environment interaction. Therefore, although three pairs *PTK2B*-*CR1*, *PTK2B-CD33*, *CD33-CR1* interaction appeared to have strong effects on LOAD, there may be unmeasured environment factors participating in these interaction. In current study, three new SNP-SNP pairs left their inter-molecular mechanism unsolved. *CD33* is a transmembrane protein which has been implicated as a negative regulator of myeloid cells. *CR1* is found on myeloid cells as well. Both of them are key molecules in inflammatory cells, such as microglia [33], therefore, we speculate they may share the similar pathway in the progression of LOAD. One AD research team from the United States now is beginning to map the molecular consequences of *CR1* and *CD33* variants to uncover their functionally link susceptibility loci [33]. With regard to *PTK2B*, which was one of recent identified new susceptible gene in 74046 individuals from diverse ethnicities [16], it was a key component of signaling pathways involved in neurite growth and synapse formation [34]. Although *PTK2B* was located on chromosome 8 near the *CLU* gene, we did not find a SNP-SNP interaction in these two genes. However, we found *PTK2B* had a strong interaction with NEDD9, which was important for lymphocyte signaling and migration and played a role in T cell-mediated inflammation [34]. Therefore, we speculate *PTK2B* might participate in immune inflammation through regulating *NEDD9* protein function, which was involved in *CR1* and *CD33*.

In summary, we examined 56 candidate SNPs in a cohort of Chinese subjects using a series of analysis methods. We identified seven risk and six protective variants for LOAD. With regard to SNP-SNP interaction, firstly, we found out some SNPs in the same gene could be influenced by each other to weaken or aggravate their single effect. Secondly, although some variants had a weak effect, a strong interaction effect was found after interacted with each other.

## Supporting Information

S1 File Table A, The PCR condition of *APOE* genotyping. (DOCX)

S2 File Table A, The details of each SNP genotyping in all subjects. (TXT)

S3 File Table A, The multivariable logistic regression analysis in identified SNPs. A multivariable logistic analysis in seven risk SNPs. Table B, A multivariable logistic regression analysis in the six protective SNPs.
(DOCX)

S4 File Table A, Identification of SNP-SNP interaction among all SNPs. 20 Candidate SNPs were selected in the first step using LMR method. Table B, Multiple linear regression model after stepwise selection.
(DOCX)
