# Supplementary material for: Polygenic Analysis of Late-Onset Alzheimer’s Disease from Mainland China
Source: PLoS One. 2015 Dec 17;10(12):e0144898. doi: 10.1371/journal.pone.0144898 (PMC4683047; doi:10.1371/journal.pone.0144898)
Supplement: S1 File — (DOCX) [file pone.0144898.s001.docx]

**Supporting information file 1**

**The PCR condition of APOE genotyping**

Table A. The PCR condition of *APOE* genotyping

| APOE: forward primer | 5’CCTACAAATCGGAACTGG3’ |
| --- | --- |
| APOE: reverse primer | 5’CTCGAACCAGCTCTTGAG3’ |

Genomic DNA was PCR amplified using a 10 μl reaction including 1μl of 50ng/μl of DNA samples, 0.8μl of dNTP mix (Takara Bio, Inc), 1μl of 10xbuffer (Takara Bio, Inc), 0.08μl of Hotstar DNA polymerase (Takara Bio, Inc), 0.5μl of 100 ng/μl of forward and reverse primer and 6.12μl MQ. The total process was performed using a touchdown thermocycling program. The reaction conditions consisted of 95°C for 5 min, then, 15 cycles of 94°C for 1 min, 62°C for 1 min with a decrement of 0.5°C per cycle, 72°C for 1 min; followed by 28 cycles of 94°C for 30 sec; 56°C for 1 min, 72°C for 1 min, and followed by 72°C for 10 min, then, the final temperature was sustained at 4°C.
