# Supplementary material for: Polygenic Analysis of Late-Onset Alzheimer’s Disease from Mainland China
Source: PLoS One. 2015 Dec 17;10(12):e0144898. doi: 10.1371/journal.pone.0144898 (PMC4683047; doi:10.1371/journal.pone.0144898)
Supplement: S3 File — A multivariable logistic analysis in seven risk SNPs. Table B, A multivariable logistic regression analysis in the six protective SNPs. (DOCX) [file pone.0144898.s003.docx]

**Supporting information file 3**

**The multivariable logistic regression analysis in identified SNPs.**

Table A. A multivariable logistic regression analysis of the seven risk SNPs

| SNPs | P*-value | OR (95%CI) |
| --- | --- | --- |
| rs9331888 | 0.016 | 1.364 (1.059-1.756) |
| rs6691117 | 0.015 | 1.401 (1.068-1.839) |
| rs4938933 | 0.026 | 1.376 (1.040-1.820) |
| rs9349407 | 0.021 | 1.454 (1.058-1.998) |
| rs1160985 | 0.101 | 1.269 (0.995-1.685) |
| rs4945261 | 0.015 | 1.382 (1.065-1.792) |
| rs5984894 | 0.000 | 1.878 (1.350-2.612) |

*Adjusting age, gender and APOEε4.

Table B. A multivariable logistic regression analysis of the six protective SNPs

| SNPs | P*-value | OR (95%CI) |
| --- | --- | --- |
| rs744373 | 0.002 | 0.665 (0.511-0.864) |
| rs1562990 | 0.002 | 0.668 (0.518-0.863) |
| rs157581 | 0.905 | 0.977 (0.664-1.436) |
| rs11556505 | 0.003 | 0.471 (0.287-0.772) |
| rs597668 | 0.016 | 0.737 (0.575-0.944) |
| rs9271192 | 0.018 | 0.702 (0.524-0.941) |

*Adjusting age, gender and APOEε4.
