# Supplementary material for: Polygenic Analysis of Late-Onset Alzheimer’s Disease from Mainland China
Source: PLoS One. 2015 Dec 17;10(12):e0144898. doi: 10.1371/journal.pone.0144898 (PMC4683047; doi:10.1371/journal.pone.0144898)
Supplement: S4 File — 20 Candidate SNPs were selected in the first step using LMR method. Table B, Multiple linear regression model after stepwise selection. (DOCX) [file pone.0144898.s004.docx]

**Supporting information file 4**

**Identification of SNP-SNP interaction among all SNPs.**

Table A. 20 Candidate SNPs were selected in the first step using LMR method

| No. | SNP | Odds Ratio |
| --- | --- | --- |
| 1 | 28834970 | 1.25 |
| 2 | 9271192 | 0.61 |
| 3 | 6656401 | 0.69 |
| 4 | 7561528 | 1.21 |
| 5 | 10793294 | 1.32 |
| 6 | 11218343 | 1.12 |
| 7 | 11767557 | 0.99 |
| 8 | 983392 | 1.20 |
| 9 | 2718058 | 1.29 |
| 10 | 5984894 | 1.92 |
| 11 | 3865444 | 1.73 |
| 12 | 3764650 | 0.92 |
| 13 | 1476679 | 0.88 |
| 14 | 3826656 | 1.65 |
| 15 | 17125944 | 1.05 |
| 16 | 11556505 | 0.65 |
| 17 | 4945261 | 0.74 |
| 18 | 8106922 | 1.21 |
| 19 | 12459419 | 1.71 |
| 20 | 1160985 | 1.38 |

Table B. Multiple linear regression model after stepwise selection

| NO. | SNP1 | SNP2 | Estimate | | Std. Error | t value | p-value | adjusted p-value |
| --- | --- | --- | --- | --- | --- | --- | --- | --- |
| 1 | (Intercept) | | | 0.34 | 0.11 | 2.99 | 2.95E-03 | 0.561 |
| 2 | 9271192 | | | 0.34 | 0.08 | 4.42 | 1.21E-05 | 0.002 |
| 3 | 5984894 | | | -0.16 | 0.04 | -4.11 | 4.74E-05 | 0.009 |
| 4 | 1160985 | | | 0.32 | 0.10 | 3.30 | 1.04E-03 | 0.198 |
| 5 | 2718058 | | | -0.24 | 0.08 | -3.21 | 1.42E-03 | 0.269 |
| 6 | 28834970 | | | -0.14 | 0.05 | -2.86 | 4.42E-03 | 0.840 |
| 7 | 4945261 | | | 0.18 | 0.06 | 2.80 | 5.35E-03 | 1 |
| 8 | 3764650 | | | 0.15 | 0.06 | 2.55 | 1.12E-02 | 1 |
| 9 | 1476679 | | | -0.13 | 0.07 | -1.86 | 6.35E-02 | 1 |
| 10 | 12459419 | | | 0.50 | 0.27 | 1.86 | 6.39E-02 | 1 |
| 11 | 11556505 | | | 0.22 | 0.12 | 1.77 | 7.75E-02 | 1 |
| 12 | 8106922 | | | -0.19 | 0.13 | -1.49 | 1.36E-01 | 1 |
| 13 | 3865444 | | | -0.39 | 0.27 | -1.43 | 1.54E-01 | 1 |
| 14 | 11218343 | | | -0.06 | 0.05 | -1.14 | 2.55E-01 | 1 |
| 15 | 3826656 | | | -0.08 | 0.08 | -0.96 | 3.37E-01 | 1 |
| 16 | 11767557 | | | -0.07 | 0.11 | -0.61 | 5.40E-01 | 1 |
| 17 | 7561528 | | | 0.04 | 0.09 | 0.44 | 6.59E-01 | 1 |
| 18 | 17125944 | | | 0.01 | 0.05 | 0.29 | 7.73E-01 | 1 |
| 19 | 983392 | | | -0.03 | 0.11 | -0.23 | 8.21E-01 | 1 |
| 20 | 6656401 | | | 0.04 | 0.19 | 0.22 | 8.24E-01 | 1 |
| 21 | 10793294 | | | 0.00 | 0.07 | 0.02 | 9.81E-01 | 1 |
| 22 | **6656401*** | **3865444** | | **-0.62** | **0.16** | **-3.96** | **8.74E-05** | **0.017** |
| 23 | **28834970** | **6656401** | | **0.64** | **0.16** | **3.94** | **9.30E-05** | **0.018** |
| 24 | **28834970** | **3865444** | | **0.27** | **0.07** | **3.79** | **1.68E-04** | **0.032** |
| 25 | 2718058 | 8106922 | | 0.24 | 0.07 | 3.65 | 2.93E-04 | 0.056 |
| 26 | 9271192 | 3764650 | | -0.18 | 0.05 | -3.55 | 4.22E-04 | 0.080 |
| 27 | 9271192 | 983392 | | 0.28 | 0.09 | 3.22 | 1.35E-03 | 0.257 |
| 28 | 10793294 | 4945261 | | 0.19 | 0.06 | 3.12 | 1.90E-03 | 0.360 |
| 29 | 7561528 | 3826656 | | 0.27 | 0.09 | 3.04 | 2.46E-03 | 0.468 |
| 30 | 11767557 | 3865444 | | -0.30 | 0.10 | -3.04 | 2.53E-03 | 0.481 |
| 31 | 11218343 | 1476679 | | 0.13 | 0.05 | 2.59 | 9.81E-03 | 1 |
| 32 | 3764650 | 4945261 | | -0.11 | 0.04 | -2.51 | 1.23E-02 | 1 |
| 33 | 9271192 | 10793294 | | -0.14 | 0.06 | -2.50 | 1.26E-02 | 1 |
| 34 | 11767557 | 1160985 | | -0.16 | 0.06 | -2.45 | 1.46E-02 | 1 |
| 35 | 4945261 | 1160985 | | -0.12 | 0.05 | -2.45 | 1.46E-02 | 1 |
| 36 | 7561528 | 12459419 | | -0.24 | 0.10 | -2.42 | 1.59E-02 | 1 |
| 37 | 11767557 | 4945261 | | 0.15 | 0.06 | 2.41 | 1.65E-02 | 1 |
| 38 | 7561528 | 1476679 | | 0.15 | 0.06 | 2.40 | 1.70E-02 | 1 |
| 39 | 9271192 | 7561528 | | -0.15 | 0.06 | -2.38 | 1.76E-02 | 1 |
| 40 | 3865444 | 1476679 | | 0.18 | 0.08 | 2.37 | 1.82E-02 | 1 |
| 41 | 28834970 | 2718058 | | 0.14 | 0.06 | 2.34 | 1.97E-02 | 1 |
| 42 | 1476679 | 3826656 | | -0.15 | 0.07 | -2.21 | 2.74E-02 | 1 |
| 43 | 10793294 | 983392 | | -0.21 | 0.10 | -2.13 | 3.37E-02 | 1 |
| 44 | 6656401 | 11556505 | | 0.37 | 0.18 | 2.09 | 3.74E-02 | 1 |
| 45 | 983392 | 3826656 | | 0.27 | 0.13 | 2.05 | 4.12E-02 | 1 |
| 46 | 983392 | 3865444 | | -0.27 | 0.13 | -2.01 | 4.49E-02 | 1 |
| 47 | 3764650 | 8106922 | | 0.17 | 0.09 | 2.01 | 4.53E-02 | 1 |
| 48 | 3764650 | 1160985 | | -0.16 | 0.08 | -2.00 | 4.63E-02 | 1 |
| 49 | 11218343 | 983392 | | -0.18 | 0.09 | -2.00 | 4.64E-02 | 1 |
| 50 | 10793294 | 11556505 | | -0.16 | 0.08 | -2.00 | 4.66E-02 | 1 |
| 51 | 11767557 | 17125944 | | -0.12 | 0.06 | -1.98 | 4.80E-02 | 1 |
| 52 | 11767557 | 11556505 | | 0.16 | 0.08 | 1.98 | 4.81E-02 | 1 |
| 53 | 28834970 | 7561528 | | -0.12 | 0.06 | -1.94 | 5.28E-02 | 1 |
| 54 | 9271192 | 3826656 | | 0.10 | 0.05 | 1.91 | 5.72E-02 | 1 |
| 55 | 3865444 | 4945261 | | -0.09 | 0.05 | -1.88 | 6.02E-02 | 1 |
| 56 | 2718058 | 1476679 | | 0.10 | 0.05 | 1.88 | 6.14E-02 | 1 |
| 57 | 6656401 | 3764650 | | -0.24 | 0.13 | -1.86 | 6.33E-02 | 1 |
| 58 | 11556505 | 4945261 | | -0.14 | 0.07 | -1.86 | 6.34E-02 | 1 |
| 59 | 5984894 | 17125944 | | 0.10 | 0.05 | 1.82 | 6.97E-02 | 1 |
| 60 | 9271192 | 11556505 | | -0.14 | 0.08 | -1.81 | 7.10E-02 | 1 |
| 61 | 1476679 | 8106922 | | 0.10 | 0.06 | 1.80 | 7.29E-02 | 1 |
| 62 | 11767557 | 3764650 | | 0.10 | 0.06 | 1.79 | 7.46E-02 | 1 |
| 63 | 9271192 | 2718058 | | -0.10 | 0.06 | -1.78 | 7.52E-02 | 1 |
| 64 | 10793294 | 1160985 | | -0.10 | 0.06 | -1.66 | 9.68E-02 | 1 |
| 65 | 11556505 | 8106922 | | 0.18 | 0.11 | 1.64 | 1.02E-01 | 1 |
| 66 | 10793294 | 11767557 | | 0.12 | 0.07 | 1.61 | 1.08E-01 | 1 |
| 67 | 28834970 | 3826656 | | -0.10 | 0.06 | -1.60 | 1.11E-01 | 1 |
| 68 | 6656401 | 4945261 | | -0.24 | 0.15 | -1.56 | 1.19E-01 | 1 |
| 69 | 3826656 | 17125944 | | -0.07 | 0.05 | -1.55 | 1.22E-01 | 1 |
| 70 | 3865444 | 1160985 | | -0.08 | 0.05 | -1.53 | 1.28E-01 | 1 |
| 71 | 11767557 | 3826656 | | 0.14 | 0.09 | 1.50 | 1.35E-01 | 1 |
| 72 | 7561528 | 2718058 | | -0.12 | 0.08 | -1.49 | 1.38E-01 | 1 |
| 73 | 983392 | 11556505 | | 0.16 | 0.11 | 1.48 | 1.39E-01 | 1 |
| 74 | 11218343 | 2718058 | | 0.08 | 0.06 | 1.47 | 1.42E-01 | 1 |
| 75 | 1476679 | 11556505 | | 0.09 | 0.06 | 1.42 | 1.57E-01 | 1 |
| 76 | 9271192 | 1476679 | | -0.07 | 0.05 | -1.38 | 1.69E-01 | 1 |
| 77 | 8106922 | 1160985 | | -0.08 | 0.06 | -1.34 | 1.82E-01 | 1 |

*Boldface represented three identified SNP-SNP pairs
